# Astrocytic modulation of neuronal signalling

**DOI:** 10.3389/fnetp.2023.1205544

**Published:** 2023-06-01

**Authors:** Sushmitha S. Purushotham, Yossi Buskila

**Affiliations:** ^1^ School of Medicine, Western Sydney University, Campbelltown, NSW, Australia; ^2^ The MARCS Institute, Western Sydney University, Campbelltown, NSW, Australia

**Keywords:** astrocytes, excitability, synapse, neuromodulation, oscillations

## Abstract

Neuronal signalling is a key element in neuronal communication and is essential for the proper functioning of the CNS. Astrocytes, the most prominent glia in the brain play a key role in modulating neuronal signalling at the molecular, synaptic, cellular, and network levels. Over the past few decades, our knowledge about astrocytes and their functioning has evolved from considering them as merely a brain glue that provides structural support to neurons, to key communication elements. Astrocytes can regulate the activity of neurons by controlling the concentrations of ions and neurotransmitters in the extracellular milieu, as well as releasing chemicals and gliotransmitters that modulate neuronal activity. The aim of this review is to summarise the main processes through which astrocytes are modulating brain function. We will systematically distinguish between direct and indirect pathways in which astrocytes affect neuronal signalling at all levels. Lastly, we will summarize pathological conditions that arise once these signalling pathways are impaired focusing on neurodegeneration.

## Introduction

Astrocytes are increasingly accepted as “Master-regulators” of brain function, mainly due to their ability to modulate various neuronal processes via direct and indirect pathways at the molecular, synaptic, cellular, and network levels (Walz, 2000; Pascual et al., 2005; Santello and Volterra, 2009; Chung et al., 2015; Anderson et al., 2016; Heithoff et al., 2021). Mounting evidence suggests that astrocytes directly modulate synaptic activity, including synaptic transmission, formation, and elimination as well as neuronal repair (Newman, 2003; Achour and Pascual, 2010; Chung et al., 2015; Burda et al., 2016). Moreover, astrocytes can affect neuronal activity indirectly via regulation of the transfer of metabolites through the blood-brain barrier, provision of metabolic support, and maintenance of ionic homeostasis in the extracellular environment (Simard and Nedergaard, 2004; Deitmer et al., 2019; Rose et al., 2020b), see also Table 1. In the below section, we have attempted to summarise the molecular signalling pathways underlying the astrocytic effect on neuronal signalling and function.

**TABLE 1:** Direct and indirect effects of astrocytes on neuronal signalling.

Molecule	Direct effect on neuronal activity	Indirect effect on neuronal activity
Ca^2+^	• Suppression of spontaneous synchronous calcium oscillations (SSCO) in neurons Berezhnov et al., 2021	• Astrocytic ATP release lead to the formation of extracellular Adenosine, which upregulates synaptic inhibition of pyramidal cells via Somatostatin^+^ cells Matos et al., 2018
	• Regulation of neuronal excitability, signal conduction and action potential modulation via Ca^2+^ dependent ATP release Lezmy et al., 2021	
	• Downregulation of synaptic and extra synaptic GABA receptors via Ca^2+^ dependent ATP release Lalo et al., 2014	
	• LTP is regulated by different astrocytic IP_3_Rs via D-serine release Sherwood et al., 2017	
Na^+^	• Modulation of excitatory and inhibitory synapses by direct uptake of neurotransmitters Todd et al., 2017; Andersen et al., 2020	• Neuronal heterosynaptic depression via astrocytic NCX channels Boddum et al., 2016
		• Regulation of neuronal [Na^+^]_i_ loads during hyper-synchronised activity Karus et al., 2015
K^+^	• Modulation of neuronal oscillations Sibille et al., 2015	• Modulation of neuronal hyperexcitability Djukic et al., 2007; Bay and Butt, (2012)
		• Modulation of synaptic plasticity Sibille et al., 2014
Glutamate	• Maintains extracellular glutamate levels and prevents neuronal hyperexcitability Sicot et al., 2017; Limbad et al., 2020	• Avert unnecessary glutamate spillover and extrasynaptic NMDAR-excitatory postsynaptic currents Trabelsi et al., 2017
	• Contributes to LTP and modulates synaptic plasticity Buskila et al., 2005; Bonansco et al., 2011; Han et al., 2013; Gómez-Gonzalo et al., 2015; Park et al., 2015; Falcón-Moya et al., 2020	• Modulation of cortical UP states and synchronous activity in astrocytic domains Poskanzer and Yuste, (2011)
GABA	• Contribution to tonic GABA inhibition Yoon et al., 2011; Yoon et al., 2014; Lee et al., 2022c	• Evokes slow inwards currents (SICs) in neurons Mariotti et al., 2016
	• Modulation of both phasic and tonic currents Lalo et al., 2014	• Homeostatic regulation of excitatory transmission Chen et al., 2013; Boddum et al., 2016

## Astrocytic modulation of neuronal signalling at the molecular level

### Ca^2+^ signalling

Ca^2+^ ions are one of the most important secondary messenger molecules in the brain. They play a key role in various signalling cascades and processes that account for both normal physiological functioning (Blaustein, 1985), as well as pathological conditions (Shigetomi et al., 2019). Although astrocytes are not capable of generating action potentials, they can communicate with coupled astrocytes and other neurons in their vicinity through transient elevations of intracellular Ca^2+^ concentration (termed Ca^2+^ oscillations or waves). Indeed these Ca^2+^ signals are referred to as the main readouts of astrocytic function (Bazargani and Attwell, 2016). Astrocytic Ca^2+^ signals affect the diffusion of ions across the astrocytic syncytium, and the release of chemical messengers (Newman, 2003; Parpura and Verkhratsky, 2012), thereby effecting both excitatory and inhibitory neurotransmission, basal synaptic activity, and synaptic plasticity directly or indirectly (Shigetomi et al., 2008; Panatier et al., 2011; Matos et al., 2018).

The role of astrocytic Ca^2+^ signalling in regulating neuronal physiology is a debatable topic within the scientific community (see (Bazargani and Attwell, 2016) for a detailed overview). On one hand, a group of studies advocated that a rise in astrocytic intracellular Ca^2+^ concentrations [Ca^2+^]_i_ leads to an increase in neuronal [Ca^2+^]_i_ indicating that synaptic activity is associated with and influenced by fluctuations in the astrocytic [Ca^2+^]_i_ (Nedergaard, 1994; Parpura et al., 1994), while on the other hand, a group of studies argued that selective stimulation of astrocytic Ca^2+^ does not increase the neuronal Ca^2+^ levels thereby having no effect on the excitatory synaptic activity (Fiacco et al., 2007; Petravicz et al., 2008). Nonetheless, several studies have emphasized the role of astrocytic Ca^2+^ in regulating neuronal activity. In primary co-culture of neurons and astrocytes, it was discovered that spontaneous synchronous calcium oscillations (SSCO) in neurons can be suppressed by dopamine-induced Ca^2+^ signals in astrocytes and could be modified by stimulation of astrocytic Ca^2+^ rise leading to the release of GABA or adrenaline (Berezhnov et al., 2021). Astrocytic Ca^2+^ also increases basal synaptic transmission by activation of pre-synaptic α-2 adrenergic receptor (AA2 receptors) (Panatier et al., 2011) and is found necessary for intact functioning of the tripartite synapses in the hippocampus (Tanaka et al., 2013).

Other studies have pointed out the crucial role of astrocytic Ca^2+^ oscillations in mediating synaptic plasticity including LTP in the hippocampus. (Gómez-Gonzalo et al., 2015; Sherwood et al., 2017). Ca^2+^modulate astrocytic release of D-serine which activates NMDARs in the vicinity and thus contributing to the induction of hippocampal LTP (Henneberger et al., 2010) implying the involvement of astrocytes in learning and memory processes, which is contradictory to earlier studies (Petravicz et al., 2008; Agulhon et al., 2010). Moreover, Ca^2+^ signals were found to serve as a bridge in converting cholinergic activity into somatosensory plasticity which is associated with sensory functions (Takata et al., 2011) and provides evidence for astrocytic-neuronal interaction during sensory information processing (Lines et al., 2020). Furthermore, Lezmy and colleagues discovered that the astrocytic Ca^2+^ mediated ATP release into the extracellular milieu plays a key role in regulating the excitability and conduction velocity of myelinated cortical axons, suggesting that astrocytic [Ca^2+^]_i_ directly controls the flow of information, neuronal signalling, and modulates action potentials (Sasaki et al., 2011; Lezmy et al., 2021). Recent studies suggested that astrocytic Ca^2+^ signalling is associated with regulating inhibitory synapses in a specific population of neurons wherein, elevated astrocytic Ca^2+^ leads to increased Somatostatin-expressing interneurons (SOM-INs) inhibition of pyramidal cells through the release of ATP (Lalo et al., 2014; Matos et al., 2018) or by endocytosis of the GABA transporter (GAT) from the plasma membrane of astrocytes (Zhang et al., 2017). Together, these studies suggest that astrocytic Ca^2+^ signalling is imperative in regulating neuronal activity, shaping the network function and thus forming a gateway for astrocytic-neuronal communication.

### Na^+^ signalling

Intracellular Na^+^ [Na^+^]_i_ transients are a fundamental property of both protoplasmic and fibroblastic astrocytes (Moshrefi-Ravasdjani et al., 2017) which represent a mechanism for fast and local signalling at the single perisynaptic level and determine the functional activity of astrocytes (Kirischuk et al., 2012). Na^+^ signals in astrocytes develop following the activation of excitatory synaptic transmission, which activates glutamate uptake into astrocytes and induces Na^+^ signals that propagate into the astrocytic syncytium via gap junctions (Langer et al., 2012; Langer et al., 2017). These signals are involved in a wide range of processes, including utilization of glutamate and lactate, K^+^ buffering, and transport of neurotransmitters (Voutsinos-Porche et al., 2003; Shimizu et al., 2007; Reyes et al., 2012; Hertz et al., 2015). Astrocytes express a plethora of Na^+^-permeable ion channels (iGluRs, ATPase’s- Na/K^+^ ATPase), exchangers and cotransporters (NKCC1; NCX; NHE; NBC, Na_x_) which are essential for developing ionic gradients required for Na^+^ signalling on the one hand, and maintain Na^+^ homeostasis on the other, reviewed by (Kirischuk et al., 2012; Rose and Karus, 2013). As most of the ionic channels that are expressed by astrocytes are also expressed by neurons, deciphering the selective impact of the different Na^+^ channels and transporters on astrocytic functioning is highly challenging.

The majority of the astrocytic Na^+^ transients affect neuronal signalling and function indirectly. One of the fundamental functions of astrocytic Na^+^ signals is to provide adequate metabolic support to neurons (neuro-metabolic coupling) for the transportation of neurotransmitters, ions, amino acids, and molecules across the membrane through the “lactate shuttle”, which is essential to form long-term memory (Bélanger et al., 2011; Langer et al., 2017; Descalzi et al., 2019). The level of various neurotransmitters such as glutamate, GABA, and glycine in the extracellular milieu is modulated by their respective transporters and enzymes whose activity is steered by astrocytic Na^+^ levels (Kirischuk et al., 2007; Héja et al., 2009; Unichenko et al., 2012), for details review see (Kirischuk et al., 2016). For example, the regulation of glutamate recycling from excitatory synapses is mediated by glutamine release from astrocytes, which is found to be modulated by elevated astrocytic [Na^+^]_i_ via the Sodium-coupled neutral amino acid transporter 3 (SNAT-3) (Uwechue et al., 2012; Todd et al., 2017). Astrocytes also govern the GABAergic transmission and metabolism by direct uptake of GABA via the Na^+^ dependant GAT3 pathway and indirectly via glutamine synthesis to sustain in neuronal terminals, indicating GABA sensitivity to the astrocytic Na^+^ dependant supply of glutamine (Ortinski et al., 2010; Andersen et al., 2017; Andersen et al., 2020).

Recently, the reversal operation of the NCX exchanger in astrocytes has gained a lot of attention as it is capable of converting Na^+^ currents to Ca^2+^ signals in the astrocytic soma, perisynaptic cradle and thin astrocytic processes and thus, can potentially regulate the excitatory and inhibitory activity of neurons as discussed above (Brazhe et al., 2018; Verveyko et al., 2019; Wade et al., 2019; Rose et al., 2020a; Héja and Kardos, 2020). In particular, the activity of GAT-3 in hippocampal astrocytes leads to an increase in [Na^+^]_i_ that translates to Ca^2+^ signals via NCX and triggers the release of ATP, which result in presynaptic inhibition of glutamate release in the adjacent neurons and heterosynaptic depression (Boddum et al., 2016). Moreover, NCX facilitates Ca^2+^-dependent glutamatergic gliotransmission at the tripartite synapse, directly affecting synaptic and neuronal activity (Reyes et al., 2012). Astrocytic Na^+^ transients also regulate the neuronal [Na^+^]_i_ loads during hyper-synchronised activity by restricting Na^+^ discharge duration through glutamate and K^+^ uptake, hence aiding neurons to recover from Na^+^ loads that are induced during epileptic activity (Karus et al., 2015). Differences in the magnitude of astrocytic Na^+^ currents in the cortex and hippocampal regions have been observed, suggesting their diverse functional roles (Ziemens et al., 2019). Interestingly, axonal glutamate-evoked astrocytic Na^+^ currents were reported in the white matter, a brain region devoid of synapses and were spread to oligodendrocytes and NG2 glia oligodendrocytes, a process termed as ‘*Panglial passage’* (Moshrefi-Ravasdjani et al., 2017). These astrocytic Na^+^ currents in the white matter can potentially translate to Ca^2+^ currents via reversal of NCX, thus speculating the indirect role of astrocytic Na^+^ currents in modulating neuronal signal conduction and propagation via translated Ca^2+^ currents (Dutta et al., 2018; Lezmy et al., 2021). Moreover, the fact that astrocytic glutamate uptake from the synaptic cleft is driven by Na^+^ currents indicates its paramount importance in defining glutamate excitotoxicity and homeostasis implicated in various neurodegenerative diseases (Li et al., 2021). In conclusion, astrocytic Na^+^ transients are important in regulating the neurotransmitter pool and affect the intracellular astrocytic Ca^2+^ levels thereby having indirect control over neuronal signalling, processing, and activity.

### K^+^ signalling

Potassium ions are critical in determining neuronal activity as neurons are extremely sensitive to K^+^ ions (Kocsis et al., 1983). Following neuronal activity, local extracellular K^+^ concentration [K^+^]_o_ increases, which leads to depolarization of both neurons (Baylor and Nicholls, 1969; Yarom et al., 1982) and glia (Orkand et al., 1966). Long-standing high [K^+^]_o_ can affect the ability of neurons to fire action potentials, transmit synaptic signals, and re-uptake of neurotransmitters. Therefore, clearance of [K^+^]_o_ from the extracellular milieu is of paramount importance for brain function. Most [K^+^]_o_ clearance in the brain is facilitated by astrocytes via various mechanisms, including ‘K^+^ uptake’ via inwardly rectifying K^+^ (K_ir_) channels (Larsen and MacAulay, 2014), Na^+^/K^+^-ATPase (NKA) and Na^+^/K^+^/2Cl^-^ (NKCC) cotransporters (Larsen and MacAulay, 2014; Sibille et al., 2015), and spatial buffering via astrocytic coupled gap junctions (Orkand et al., 1966; Ransom, 1996; Ma et al., 2016) which underscores the role of astrocytes in maintaining K^+^ homeostasis.

Astrocytic K^+^ signalling is involved in K^+^ and glutamate homeostasis (Djukic et al., 2007; Kucheryavykh et al., 2007), regulation of neuronal oscillations (Bellot-Saez et al., 2018), neural rhythms, hyperexcitability, synaptic plasticity, and locomotor behaviour (Bellot-Saez et al., 2017; Kelley et al., 2018; Barbay et al., 2023). Retrieval of K^+^ from the extracellular milieu is essential to prevent pathological accumulation of K^+^ in the extracellular space (Ransom, 1996; Bellot-Saez et al., 2017; Bellot-Saez et al., 2021), if not can result in depolarization of nearby neurons that affects their excitability profile (Do-Ha et al., 2018). By carrying out specific knock-out, pharmacological, and genetic inhibition of astrocytic K_ir_4.1 channels, Djukic and colleagues reported an increase in [K^+^]_o_, resulting in astrocyte’s inability to retrieve K^+^ and glutamate at the tripartite synapse, making the neurons vulnerable to hyperexcitable states (Djukic et al., 2007; Bay and Butt, 2012; Tong et al., 2014; Tyurikova et al., 2022) that is often observed in pathological conditions (Bellot-Saez et al., 2017). Moreover, increased [K^+^]_o_ hampered glutamate uptake and led to a reduction in mEPSC frequency, indicating a negative feedback system which caused suppression of basal excitatory neurotransmission during the physiological state (Rimmele et al., 2017). By modelling the tripartite synapse and performing electrophysiological recordings, it was shown that K_ir_4.1 channels remarkably contribute to bringing the K^+^ and neuronal excitability to basal levels, particularly in response to repetitive stimulation and mainly modulates the neuronal theta rhythmic activity (Sibille et al., 2015). Moreover, K_ir_ 4.1 conditional KO mice experience an increase in the LTP (Djukic et al., 2007), and a subsequent study provided evidence that astrocytic K_ir_4.1 channels suppress short-term synaptic plasticity responses specifically induced by prolonged repetitive stimulation and post-tetanic potentiation (PTP). (Sibille et al., 2014).Taken together, astrocytic K^+^ clearance is vital for shaping neuronal activity at both cellular and network levels as well as behaviour.

### Glutamate signalling

Glutamate is one of the most abundant amino acids in the brain and owing to its property as an excitatory neurotransmitter, it is essential to restrict its availability within the synaptic cleft, as excess glutamate leads to *excitotoxicity* and neuronal death (Curtis and Johnston, 1974; Fonnum, 1984; Buskila et al., 2005; Dong et al., 2009). Approximately 80% of glutamate is retrieved from the synaptic cleft by astrocytes via glutamate transporters, namely, glutamate-aspartate transporter (GLAST), glutamate transporter-1 (GLT-1) (Rothstein et al., 1995; Rothstein et al., 1996), and excitatory amino acid transporters (EAATs) (Magi et al., 2019). Astrocytes actively act as scavengers of glutamate by increasing the surface diffusion of affinity glutamate transporters (Al Awabdh et al., 2016). The senescence of astrocytes is linked to cortical neuronal excitability due to heightened glutamate toxicity (Limbad et al., 2020). Computational studies suggest that any elevations in astrocytic glutamate concentrations can retain the glutamate in the synaptic cleft for longer periods and thus lead to an increased magnitude of slow inward currents (SICs), potentially resulting in hyperexcitability (Li et al., 2016a; Flanagan et al., 2018). Therefore, the activity of glutamate transporters in astrocytes is strictly regulated by transmembrane Na^+^ concentrations, that indirectly contribute to shaping synaptic transmission (Verkhratsky and Steinhäuser, 2000; Lalo et al., 2011). Particularly, Bergmann glial (BG) GLAST is indispensable for the excitatory synaptic wiring and wrapping of Purkinje cells in the cerebellar cortex (Miyazaki et al., 2017) and its reduced expression causes increased Purkinje cell firing, hyperactivity, and subsequent loss of Purkinje cells contributing to myotonic dystrophy and spinocerebellar ataxia type 1 (SCA1) (Cvetanovic, 2015; Sicot et al., 2017). The glutamate taken up is converted into glutamine-a precursor for glutamate and GABA synthesis by the action of the astrocytic enzyme glutamine synthetase (GS) and transported to neurons via Na^+^-coupled neutral amino acid transporters (SNATs) and their release is driven by astrocytic intracellular Ca^2+^ concentration. This conversion is vital to avert glutamate spill over events and unnecessary peri/extrasynaptic NMDAR-excitatory postsynaptic currents in pyramidal cells (Trabelsi et al., 2017). The glutamate uptake kinetics in astrocytes varies significantly from neonatal to adult and exhibits regional heterogeneity, explaining the possible specialized functions of astrocytes that are circuit-specific (Hanson et al., 2015; Romanos et al., 2019). This astrocytic glutamate-neuronal signalling accounts for synaptic plasticity, contributes to the synchronous activity in neuronal domains and modulates the cortical UP states by tuning the spatiotemporal levels of glutamate in the extracellular space (Fellin et al., 2004; Carmignoto and Fellin, 2006; Bonansco et al., 2011; Poskanzer and Yuste, 2011). Interestingly, astrocytic glutamate signalling also contributes to synaptic plasticity and learning and memory processes by glutamate uptake during early and late LTP (Pita-Almenar et al., 2012), as well as release of glutamate to induce NMDAR mediated LTP (Han et al., 2013; Gómez-Gonzalo et al., 2015; Park et al., 2015). It further contributes to the developmental transition from spike timing-dependent long-term depression (t-LTD) to t-LTP in the CA3-CA1 synapses of the postnatal hippocampus, thus highlighting the importance of astrocytic glutamate signalling in regulating neuronal activity from postnatal to mature stages of development (Falcón-Moya et al., 2020).

### GABA signalling

Astrocytes express a plethora of GABA receptors and transporters, including the ionotropic GABA_A_ and metabotropic GABA_B_ receptors (Fraser et al., 1994; Oka et al., 2006), and the GAT-1 and GAT-3 transporters, which facilitate GABA uptake from the synaptic cleft as part of the Glutamate/GABA-glutamine cycle (Ribak et al., 1996; Yan and Ribak, 1998; Boisvert et al., 2018; Ghirardini et al., 2018). Astrocytes are also capable of releasing a considerable amount of GABA (Lee et al., 2010b; Le Meur et al., 2012) and regulates its concentration in the extrasynaptic space (Schousboe et al., 1977). Similar to glutamate, GABA evokes Ca^2+^ oscillations in astrocytes (Meier et al., 2008) which are mainly mediated by either GABA receptors or GAT transporters. These oscillations lead to the release of GABA, glutamate, and ATP which can regulate and modulate local synaptic activity in several ways, as discussed below (Le Meur et al., 2012; Oh et al., 2012; Shlosberg et al., 2012; Boddum et al., 2016; Mariotti et al., 2016).

Recent studies indicated that GABA signalling in astrocytes can affect both the excitatory and inhibitory activity of neurons emphasising the dual role astrocytes play in network activity. Indeed, Mariotti and colleagues, recently showed that GABA-activated astrocytes release glutamate, which in turn triggered slow inward currents (SICs) and firing of nearby pyramidal neurons (Mariotti et al., 2016). Moreover, astrocytic GABA signalling was found to be involved in the homeostatic regulation of excitatory transmission by activation of astrocytic GAT-3, which on the one hand triggers Ca^2+^ mediated release of ATP that hinders presynaptic glutamate release, and on the other hand is required for heterosynaptic depression (hLTD) that is usually accompanied during LTP (Chen et al., 2013; Boddum et al., 2016).

GABA-activated astrocytes are also involved in regulating the inhibitory activity of neurons, as activation of somatostatin-expressing interneurons (SOM-INs) can trigger astrocytic GABA_B_ receptor and GATs activity to induce synaptic depression in pyramidal cells (Shlosberg et al., 2003; Shlosberg et al., 2012; Matos et al., 2018; Shen et al., 2022). It is important to note that astrocytes can also synthesise GABA through various enzymes and pathways, namely, GABA synthetase or glutamic acid decarboxylase (GAD67 or GAD65) (Chattopadhyaya et al., 2007; Walls et al., 2010), monoamine oxidase B (MAOB) involving putrescine (Yoon et al., 2014), and diamine oxidase (DAO) (Kim et al., 2015). The direct effect of astrocytic GABA on neurons was reported by Yoon *et al.*, where they showed that the amount of GABA released by astrocytic Best1 channels directly correlates to the magnitude of tonic inhibition in several brain areas including the cerebellum, thalamus, and dentate gyrus (Yoon et al., 2011). Nevertheless, it is obscure whether astrocytic synthesis of GABA alone is enough for its release and exerts a direct effect on neurons. To evaluate this, Lee and colleagues generated astrocyte-specific MAOB conditional knockout mice and found a decrease in MAOB and astrocytic GABA levels in the cerebellum and striatum which contributed to a 74%–76% reduction of tonic GABA currents in neurons, confirming that astrocytic source of GABA contributes to tonic GABA currents (Yoon et al., 2014; Lee et al., 2022c). Additionally, astrocytes of the dorsal horn also release GABA in response to glutamate suggesting their role in sensory information processing (Christensen et al., 2018). Moreover, astrocytic tonic GABA inhibition of lemniscal synapses in the thalamus can increase temporal fidelity and thus improve tactile discrimination (Kwak et al., 2020). Astrocytes also maintain the extracellular GABA concentration and are accountable for the modulation of both tonic and phasic GABA currents (Lalo et al., 2014).

Lastly, it is important to mention that GABA-activated and GABA-releasing astrocytes are capable of shifting the inhibitory signals to excitatory signals and *vice versa* through diverse mechanisms. For instance, Heja and colleagues described one of the mechanisms wherein, upon the intense excitatory activity of the neurons, astrocytes uptake glutamate which leads to the synthesis of GABA from polyamine putrescine and releases GABA via reverse operation of GAT-1/3 that result in neuronal tonic inhibition (Héja et al., 2012). In contrast, by using GABA_B_ receptor conditional knockout mice (GB1-cKO mice) specifically in astrocytes, Perea and colleagues reported that Ca^2+^ events initiated by astrocytic GABA_B_ receptors contribute to interneuron induced synaptic potentiation via activation of mGluRs (Perea et al., 2016). A plausible explanation for this dual role of astrocytes is that astrocytic response is usually specific to neuronal inputs. For example, the intensity of interneuron firing decides the type of gliotrasmitter released by astrocytes (glutamate, ATP, GABA) that acts at the pre-synapse either resulting in potentiating or inhibiting the neurotransmitter release. This clearly demonstrates the differential control of synaptic transmission by astrocytes (Covelo and Araque, 2018). In summary, astrocytic GABA signalling regulates the excitatory and inhibitory activity of neurons and controls information processing via modulation of local network activity.

## Astrocytic regulation of neuronal signals at the synaptic and cellular levels

**FIGURE 1:**
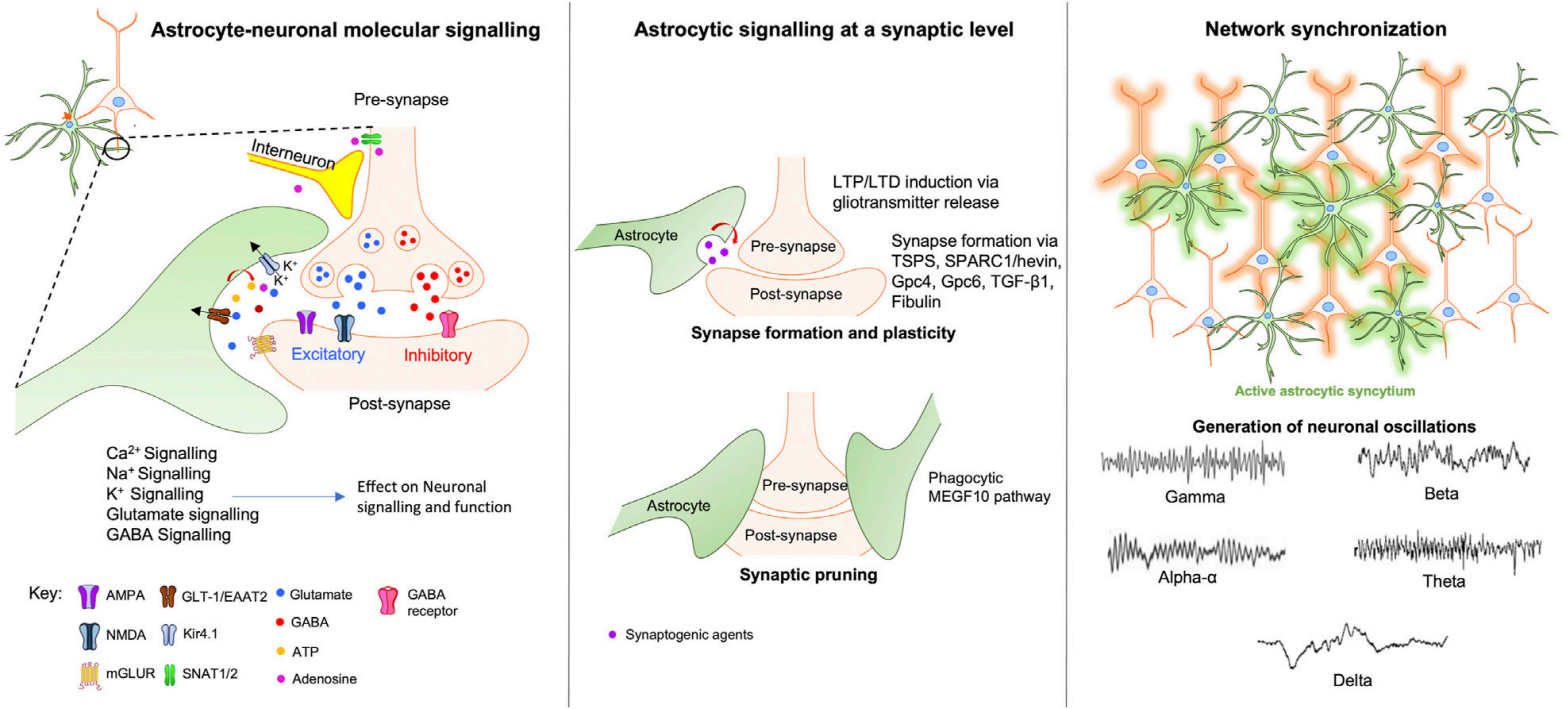
*Astrocytic modulation of neuronal signalling at the molecular, synaptic and network levels.* At the molecular level (left window) astrocytes regulate and modulate neuronal activity via various direct and indirect mechanisms involving Ca^2+^, Na^+^, K^+^, Glutamate, and GABA signalling pathways. At the synaptic level (middle window), astrocytes maintain synaptic integrity by modulating synaptic plasticity, formation, and pruning through the release of neurotransmitters, gliotransmitters and synaptogenic agents. Right window - neuronal network coordination and synchronization require the activation of astrocytic syncytium which leads to the generation of neuronal oscillations that forms the basis of various complex behaviours ranging from sleep-awake states to complex higher-order cognitive functions. Abbreviations: TSPS-Thrombospondins; SPARC1-secreted protein acidic enriched in cysteine like-1; Gpc4-glypicans 4; Gpc6-glypicans 6; TGF-β1-transforming growth factor-beta; MEGF10-Multiple EGF-like-domains 10.

The previous sections emphasised the astrocytic influence on neuronal function at a molecular level. However, mounting evidence suggests that astrocytes can also affect neuronal signalling at synaptic and cellular levels, modulating synapse formation, function and communication with other neurons and glia (Figure 1).

### Synapse formation, pruning and refinement

Since the conceptualization of the *“Tripartite Synpase”* (Araque et al., 1999) the role of astrocytic function has evolved from being mere neuroglial cells that nourish the neurons to a key player in the formation, elimination, integration, and stabilization of synapses (Chung et al., 2015). Indeed, multiple studies have shed light on the essential role of astrocytes during neuronal differentiation, formation, and maturation of synapses (Klapper et al., 2019; Van Horn and Ruthazer, 2019). Astrocytes do so via various mechanisms, including the secretion of synaptogenetic and neurotrophic factors (Baldwin and Eroglu, 2017), dynamic changes in their morphology (Lawal et al., 2022), and through uptake and release of neurotransmitters (Buskila and Amitai, 2010).

Neuronal circuits are constant shapeshifters, mainly due to synaptic plasticity processes that strengthen or weaken synapses according to one’s environmental experience. A Plethora of factors are involved in the formation and pruning of synapses. Astrocytes promote the formation and function of both excitatory (Eroglu et al., 2009) and inhibitory (Elmariah et al., 2005) synapses via the secretion of molecules that target both the pre and post-synaptic sites. Thrombospondins (TSPs) (Christopherson et al., 2005), hevin (Kucukdereli et al., 2011; Risher et al., 2014), and transforming growth factor-beta 1 (TGF-β1) (Diniz et al., 2014) are some of the major molecules secreted by astrocytes that are involved in the formation of synapses. It is interesting to note that the factors secreted by astrocytes and aid in synaptogenesis are pathway and neuron-specific and thus determine whether they become silent or active synapses. For example, thrombospondins have been associated with establishing silent glutamatergic synapses (Christopherson et al., 2005), while hevin found to be involved in synaptic refinement, particularly in thalamocortical synapses (Risher et al., 2014), and TGF-β1 found to be associated with the formation of both excitatory and inhibitory synapses (Diniz et al., 2012; Diniz et al., 2014). Complementary to this, glypicans 4 and 6 (Gpc4 and Gpc6) secreted by astrocytes found to be involved in the formation of active functional synapses (Allen et al., 2012; Farhy-Tselnicker et al., 2017). Moreover, a recent report indicated that astrocyte-derived small extracellular vesicles (SEVs) contain a synaptogenic cargo called Fibulin-2 which contributed to cortical dendritic spine and synapse formation in primary cortical neuronal cultures (Patel and Weaver, 2021). Subsequently, astrocytic lipid metabolism and mitochondrial biosynthesis are also found to play a critical role in synapse formation and maturation. The astrocytic lipid metabolism involves Sterol regulatory element binding proteins (SREBPs) whose activity is dependent on sterol sensor SREBP cleavage-activating protein (SCAP). Selective inactivation of astrocytic SCAP-SREBP-mediated lipid biogenesis led to impaired function of the pre-synaptic terminal, and short and long-term plasticity in the SCAP mutant mice (van Deijk et al., 2017). Likewise, astrocytic Fatty acid binding protein 7 (FABP7) is essential for normal dendritic morphology and miniature excitatory postsynaptic currents (mEPSCs) indicating the implication of astrocytes in regulating the excitatory synaptic function (Ebrahimi et al., 2016). The astrocytic mitochondrial biosynthesis is reliant on the metabolic regulator peroxisome proliferator-activated receptor gamma (PPARγ) co-activator 1α (PGC-1α) whose activity is governed by mGluR_5_. Conditional genetic ablation of PGC-1α led to abberant astrocytic proliferation and maturation, resulting in disrupting synaptogenesis and the excitatory synapse formation (Zehnder et al., 2021).

Apart from contributing to the formation of synapses, astrocytes also regulate the functions of synapses and take part in the process of synaptic pruning (Papouin et al., 2017; Luo and Gao, 2021). A recent study revealed that astrocytes phagocytose excitatory synapses in the hippocampus via the Multiple EGF-like-domains 10 (MEGF10) pathway and thus are accountable for replenishing memory traces via synapse elimination, which is essential for circuit homeostasis and synaptic connectivity (Lee et al., 2021). In parallel, the same astrocytic MEGF10 phagocytic receptor is responsible for the removal of thalamocortical synapses associated with ocular dominance plasticity (ODP), thus determining the synaptic plasticity that is experience-dependent (Lee et al., 2022b).

Contrary to the popular notion that the formation of synapses is involved in learning and memory, a study by Morizawa *et al* suggests that engulfment of the synapses by cerebellar Bergmann glia might result in enhanced motor learning and circuit refinement (Morizawa et al., 2022). Essentially astrocytes also produce extracellular matrix proteins and cell adhesion molecules that facilitate the integration of astrocytic processes to synapses to perform their respective functions (Hillen et al., 2018). On the whole, astrocytes are an integral part of the synapse and a key player regulating its functionality through various processes.

## Astrocytic regulation of neuronal signals at the network and behavioural levels

### Astrocytes influence neural synchrony, network oscillations, and behaviour

Neuronal synchronization and oscillations form the basis of several behaviours such as motor skills, (Davis et al., 2012), sleep-wake cycles (Adamantidis et al., 2019), and cognition (Kucewicz et al., 2014; Buskila et al., 2019a). In this section, we will highlight the importance of the astrocytic syncytium in maintaining neuronal synchronous activity that leads to the generation of synchronized oscillatory brain rhythms that underlie such behaviours.

### Astrocytic-mediated neuronal synchrony

Neuronal synchronization occurs when two or more events associated with diverse aspects of neuronal activity appear at the same time. This mainly arises due to the dynamic interplay between neurons within a network and there are several mechanisms which explain how a population of neurons get synchronized (Timofeev et al., 2012). It is suggested that astrocytic Ca^2+^ signalling corresponds to the level of neuronal synchrony in neighbouring neurons (Sasaki et al., 2014), implying that astrocytic [Ca^2+^]_i_ orchestras and maintains a collective of neuronal dynamics. Indeed, an *in vivo* study found that astrocytic Ca^2+^ regulates cortical state switching by actively regulating the extracellular glutamate levels, consequently accounting to slow neuronal rhythm thereby controlling the neural circuit states (Poskanzer and Yuste, 2016). Additionally, it has been reported that the absence of astrocytes in neuronal cultures results in desynchronized glutamate transmission that leads to perturbations in the action potential waveform and propagation (Sobieski et al., 2015).

Astrocytes facilitate various behaviours via neuronal synchrony. For example, synchrony of the neurons in the anterior cingulate cortex (ACC), a region responsible for visceral-pain-cognitive interactions is due to the astrocytic release of L-lactate, resulting in improved decision-making (Wang et al., 2017). In addition, the circadian synchrony of the suprachiasmatic nucleus (SCN) is maintained via astrocytic control of extracellular glutamate level, which is essential for normal molecular timekeeping (Brancaccio et al., 2017). Moreover, Sardinha and colleagues reported desynchronised theta oscillations between the dorsal hippocampus and the prefrontal cortex in a dominant negative SNARE (dnSNARE) mouse model with impaired exocytosis in astrocytes. They also observed reduced cognitive performances which were restored upon supplementation with D-serine (Sardinha et al., 2017), corroborating astrocytes are capable of modulating far neuronal networks, which elucidates their impact on remote synchronization activity. Furthermore, astrocytic synchronisation is preceded by neuronal synchronization, pointing to the involvement of neurons in Slow wave activity (SWA) that is associated with quite wakefulness and sleep states and emphasises that astrocytes are implicated in the generation of SWA and regulates the switching between sleep and wakefulness states, suggesting their role in sleep-wake cycle and memory consolidation (Szabó et al., 2017; Bojarskaite et al., 2020). Astrocytes also generate rhythms in the SCN and controls their period bidirectionally although to a lesser extent than neurons. Despite this, their activation does not affect the phase of the SCN in any way (Patton et al., 2022).

The regulation of neuronal synchrony by astrocytes is additionally underpinned by several neuronal-astrocytic computational models. By employing different astrocyte-neuronal simulation models, Amiri *et al* showed that astrocytes can potentially modulate the synchronous and asynchronous states of neurons by adjusting the threshold value of transition (Amiri et al., 2013). More recently, a simulation model revealed that the degree of neuronal output synchronisation increases with neuron-astrocytes interactions (Pankratova et al., 2019). Interestingly, a study employed neuronal cultures grown on a multielectrode array to explore the mechanism and origin of synchronised burst (SB) activity along with the application of computational modelling and has implicated the role of astrocytes in the generation reverberating activities in an SB (Huang et al., 2017). Similarly, astrocytic Ca^2+^ is predicted to be involved in the synchronised activity of large-scale neuronal ensembles (Li et al., 2016b; Polykretis et al., 2018) and can also cause intermittent neuron synchrony via slow astrocytic Ca^2+^ oscillations (Makovkin et al., 2020). In summary, both experimental and simulation studies suggest that astrocytes play a vital role in neuronal synchronization.

### Astrocytic modulation of neuronal oscillations and behaviour

In a given network of neurons, the synchronised activity of individual neurons leads to rhythmic variations in membrane potential, which contributes to the generation of neuronal oscillations and brain waves. These neuronal oscillations have a characteristic power and frequency band and are categorised with distinct frequency bands that are linked with particular behaviours (Buzsáki and Draguhn, 2004). Several mechanisms underlie neuronal oscillations, which include astrocytic Ca^2+^ activity (Poskanzer and Yuste, 2011; Poskanzer and Yuste, 2016), uptake and homeostasis of glutamate and potassium ions (Li et al., 2016a; Bellot-Saez et al., 2018), astrocytic coupling, and release of gliotransmitters (Pirttimaki et al., 2017).

Neuronal gamma oscillations (30–80 Hz) are associated with learning, memory and cognitive functions (Thompson et al., 2021; Liu et al., 2022) and astrocytes have been implicated in their generation and modulation. S100B, a calcium-binding protein expressed explicitly in astrocytes, is secreted to the extracellular space and contributes to the enhanced amplitude of gamma oscillations in the hippocampus (Sakatani et al., 2008). Moreover, a recent study showed that infusion of S100β into astrocytes and activation of astrocytes using Designer Receptors Exclusively Activated by Designer Drugs (DREADD) in the medial pre-frontal complex (mPFC) of rats, contributed to a rise in phase-amplitude coupling between theta and gamma oscillations and led to improved performance in the attentional set-shifting task (ASST), indicating the astrocytic role in the enhancement of cognitive flexibility (Brockett et al., 2018). Additionally, astrocytes are also known to affect goal-directed behaviours and cortical information processing, as demonstrated through genetic ablation of astrocytic GABA_B_ receptors in the mPFC, which caused a reduction in the power of low-gamma oscillations, and poor performance in goal-directed behaviour test (Mederos et al., 2021). Furthermore, lee and colleagues created a triple transgenic mouse where the astrocytic vesicular release can be reversibly controlled by the expression of tetanus neurotoxin (TeNT) in astrocytes. They observed blockade in glutamate release, shortened gamma oscillatory activity, reduction in EEG power, and impaired behaviour in novel object recognition test in these mice, indicating that astrocytes are involved in the maintenance of gamma oscillatory activity and are required for recognition memory (Lee et al., 2014).

Uptake and release of glutamate is dependent on [K]^+^_o_ which is closely regulated by astrocytes, indicating its indirect control over neuronal oscillations (Sarantis and Attwell, 1990; Longuemare et al., 1999; Djukic et al., 2007). In line with this, *in vitro* experiments in the somatosensory cortex revealed that occluding K^+^ uptake via K_ir_4.1 channels or blockade of astrocytic gap junction connectivity impairs K^+^ clearance by astrocytes and thus contributes to increased excitability of the neuronal network, leading to changes in the oscillatory behaviour of individual neurons and an increase of the network oscillatory power, thereby unravelling a novel mechanism to fine-tune neuronal network oscillations (Bellot-Saez et al., 2018). Subsequently, a recent study investigated the effect of astrocytic decoupling on network activity by generating a double KO of connexin 30 (Cx30) and connexin 43 (Cx43), where they reported a reduction in excitability in CA1 pyramidal neurons, reduced LTP, disruption of D-serine homeostasis which led to pronounced spatial memory and learning impairment (Hösli et al., 2022). Additionally, Kelley *et al* demonstrated that astrocytic K_ir_ 4.1 channels in the spinal cord are essential to induce and maintain muscle peak strength by fast alpha motor neurons, indicating that astrocytes impact the electrophysiological properties of alpha motor neurons and overall locomotor activity (Kelley et al., 2018). Moreover, dysfunctional astrocytic K_ir_4.1 channels in the spinal central pattern generator led to perturbed locomotor patterns and neuronal rhythmogenesis (Barbay et al., 2023) and it has been suggested that elevated cortical [K^+^]_o_ can impact sensory and motor processing by altering neural activity, pointing to an astrocytic role in steering such behaviours (Rasmussen et al., 2019). Another potential mechanism in which astrocytes modulate neuronal oscillations has been suggested by (Shibasaki et al., 2014), who showed that transient receptor potential vanilloid 4 positive (TRPV+) astrocytes can release gliotransmitters, namely, glutamate and ATP, which regulates the excitability of neurons. In the recent decade, much focus has been laid down on the astrocytic regulation of various behavioural paradigms, including higher-order cognitive functions such as learning and information processing (Santello et al., 2019), neuropathic and chronic pain (Li et al., 2019), motor skills (Padmashri et al., 2015), anxiety (Abu-Ghanem et al., 2008; Shim et al., 2019) and sleep (Ingiosi and Frank, 2022).

Astrocytes are involved in both short-term and long-term memory formation. For instance, mice performance was found to be reduced in spontaneous alternation Y-maze and novel object replacement task when astrocytic Gq-GPCRs were specifically blocked, indicating an impaired working and short-term spatial memory (Nagai et al., 2021). Moreover, recent fear memory, a form of long-term memory was found to be impaired when muscarinic acetylcholine receptor M1 (m1-AChRs) was specifically deleted in astrocytes from the dentate gyrus (Li et al., 2022). It is interesting to note that astrocytic metabolism is required for learning activities. A study by Descalzi *et al* reported that astrocytic lactate is critical for *de novo* mRNA translation that is induced due to learning in both excitatory and inhibitory neurons and the same was confirmed by 3D electron microscopy studies suggesting astrocytic L-lactate serves as an energy store for synaptic plasticity (Descalzi et al., 2019; Vezzoli et al., 2020). Additionally, astrocytes are involved in regulating the reward system in the brain, where they can successfully encode the location of the reward in a spatial context only in a familiar environment indicating their indirect involvement in higher cognitive functions (Doron et al., 2022). The Central nucleus of amygdala (CeA) is a region that regulates fearful and stressful responses. Studies from Knockdown of astrocytic glucocorticoid receptors (GR) in mice specifically in CeA region showed that astrocytic GR is significantly involved in consolidation of aversive memory and few anxiety-related behaviours (Tertil et al., 2018; Wiktorowska et al., 2021).

Astrocytes are also capable of reversing chronic pain and modulating motor behaviour. Indeed, allodynia-like behaviour was reversed by astrocytic initiation of spine plasticity that eliminated those synapses formed shortly after partial sciatic nerve ligation, thus affecting the circuitry implementing this information (Takeda et al., 2022). Moreover, astrocytic calcium signalling was found to be imperative for motor skill learning (Padmashri et al., 2015) and for closure of the motor circuit critical period by invading the neuropil and affecting spine dynamics, dendritic length, and synaptic inputs (Ackerman et al., 2021).

Astrocytes regulate sleep via Ca^2+^ activity and by release of adenosine which affects the sleep-wake states and sleep deprivation states (Halassa et al., 2009; Florian et al., 2011). In particular, Rapid-eye movement (REM) sleep and its associated theta rhythm is modulated by astrocytic IP3/Ca^2+^ signalling (Foley et al., 2017). Indeed, the amount of sleep corresponds to the changes in astrocytic Ca^2+^ activity and reduced intracellular Ca^2+^ levels hamper homeostatic sleep response post-sleep deprivation (Ingiosi et al., 2020). Moreover, Vaidyanathan and colleagues observed that the duration and depth of non-rapid eye movement (NREM) sleep is determined by astrocytic G-protein coupled receptor signalling pathways, namely, G_i_-GPCR (sleep depth) and G_q_GPCR (sleep duration) elucidating the contribution of network cortical astrocytes in modulating the sleep length and quality (Vaidyanathan et al., 2021). Overall, astrocytes play a critical role in regulating several behavioural attributes, which makes it extremely important to study them in greater depth.

## Astrocytic modulation of neurodegeneration

Glial cells play a key role in the mechanisms which dispose the aged brain to neurodegeneration, especially as ion homeostasis is a function that is primarily carried out by glial cells. There is a growing number of reports pointing to the perturbations in astrocytic function as a risk factor, causation, and driving factor for neurodegenerative diseases such as Alzheimer’s disease (AD), Parkinson’s Disease (PD), Huntington’s disease (HD), and Amyotrophic lateral sclerosis (ALS), all of which are associated with the loss of a specific type of neurons confined to a particular region (Stevenson et al., 2020). In this section, we will be summarising the latest studies which uncover novel astrocytic mechanisms that contribute to neurodegeneration and how targeting them can be beneficial in improving disease progression and treatment.

### Alzheimer’s disease (AD)

AD is one of the most common types of dementia and is characterised by the accumulation of extracellular *ß*-Amyloid (Aβ) plaques, intracellular neurofibrillary tangles consisting of hyperphosphorylated tau, neuronal loss, and high levels of reactive astrocytes (Ferri et al., 2005; Simpson et al., 2010; Buskila et al., 2013; Morley et al., 2018). Neurons are considered a primary repository of Aβ and apolipoprotein E4 (apoE4), a cholesterol transport protein that is considered one of the greatest risk factors for sporadic AD (Corder et al., 1993). However, a recent study has linked apoE, Aβ, and plaque formation suggesting that the production of beta-amyloid in neurons is strictly regulated by astrocytic cholesterol signalling, where apoE transports neuronal amyloid precursor protein (APP) across neuronal cell membranes using astrocyte-derived cholesterol, indicating that astrocytes are an indirect risk factor along with neuronal dysfunction (Wang et al., 2021).

The Aβ plaques are surrounded by reactive astrocytes which can either aggravate or ameliorate the disease progression. A recent study revealed that reactive astrocytes are present in the vicinity of Aβ plaques and tend to engulf, internalise, and degrade axonal dystrophic neurites associated with these plaques in both AD mouse models and patient samples (Gomez-Arboledas et al., 2018). This phagocytic nature of astrocytes indicates their involvement in clearing damaged neuronal circuits or even reducing the neuroinflammatory impact to limit the pathology of AD. Recently, Lee *et al* identified a specific population of astrocytes called autophagy-dysregulated astrocytes (APDAs) that have lost their ability to secrete synaptogenic factors and thus synapse elimination in AD and aged brain, which might be implicated in disease pathology (Lee et al., 2022a). Moreover, genetic ablation of proliferating reactive astrocytes in double APP23/GFAP-TK mice led to an increased aggregation of monomeric Aβ, which was accompanied by a reduction in synaptic and neuronal density, upregulation of pro-inflammatory markers such as TNFα, NFκB, nitric oxide synthase-1 (NOS1), and memory loss (Katsouri et al., 2020). In contrast, severe reactive astrocytes were accountable for exacerbating AD progression via H_2_O_2_ production and nitrosative stress contributing to neurodegeneration. It is interesting to note that only severe reactive astrocytic phenotype contributed to neurodegeneration whereas mild reactive astrocytic phenotype was reversible (Chun et al., 2020). In line with this study, a recent report used transcriptomic methods to show that astrocytes are equally capable of acquiring both neuroprotective as well as harmful states in Aβ and tau pathologies (Jiwaji et al., 2022). Indeed, modulation of astrocytic reactivity via the JAK2-STAT3 pathway led to reduced amyloid load and improved spatial learning (Ceyzériat et al., 2018), while specific deletion of STAT3 in the APP/PS1 mice model of AD alleviated AD symptoms such as spatial learning and memory impairments, indicating that targeting reactive astrocytes holds therapeutic benefits (Assefa et al., 2018; Reichenbach et al., 2019). Further evidence for dysregulated astrocytic Ca^2+^ (Brawek and Garaschuk, 2014) and glutamate homeostasis in the AD brain is reported as leading to hyperexcitability and neuronal death, which can be correlated to reduced GLT-1 expression and significant cognitive deficits (Brymer et al., 2023). In addition, a recent study reported regional differences in the astrocytic glutamine-glutamate cycle and impairment of synaptic mitochondrial functions in the early stages of AD which might play a key role in disease pathogenesis and progression (Andersen et al., 2021).

### Parkinson’s disease (PD)

The second most common neurodegenerative disease across the globe is PD (De Lau and Breteler, 2006). Its symptoms include tremors, rigidity, bradykinesia and other non-motor symptoms (Sveinbjornsdottir, 2016). The specific loss of dopaminergic (DA) neurons in the substantia nigra pars compacta (SNpc) and aggregation of insoluble, misfolded *a*-synuclein protein is central to the disease pathology (Hurtig et al., 2000; Kordower et al., 2013), however recent studies showed that atrophy of astrocytes, as well as astrocytic dysfunction, play a significant role in disease development (Ramos-Gonzalez et al., 2021).

During disease progression, astrocytes transform from neuroprotective to neurotoxic signatures which exacerbate the disease. The misfolded *a*-synuclein released by neurons is taken up by astrocytes via endocytosis and triggers a pro-inflammatory response (Lee et al., 2010a; Rannikko et al., 2015), including the secretion of pro-inflammatory and neuroinhibitory factors (Kekesi et al., 2019). Astrocytic response to *a*-synuclein is correlated to elevated levels of the mammalian homologue of UNC-18 (Munc18) -a protein essential for vesicle exocytosis, accompanied by reactive astrocytic morphology and increased expression of IL-6 (Di Marco Vieira et al., 2020). Indeed, Cavaliere and colleagues demonstrated that Lewy body fractions containing human α-synuclein are taken up more efficiently by astrocytes rather than neurons and induces high expression of endogenous α-syn while the transport of α-synuclein takes place bi-directionally between both cell types and causes astrogliosis (Cavaliere et al., 2017). Moreover, some of the key symptoms widely expressed in PD brains, including impaired glutamate homeostasis and signalling, and neuronal hyperexcitability (Ferrarese et al., 2001; Iovino et al., 2020; Campanelli et al., 2022) have been observed in astrocytic-specific knockdown of glutamate transporter-1 (GLT-1) in the striatum and SNpc (Zhang et al., 2020; Ren et al., 2022). A recent seminal study indicated that in PD mice, α-synuclein promotes astrocytes to produce excess glutamate, which causes synaptic loss via increased tonic glutamatergic activation of extrasynaptic NMDARs, implying the direct role astrocytes play in dysregulated glutamate signalling in PD (Trudler et al., 2021). This finding is further supported by a recent study indicating dysregulation of astrocytic Ca^2+^ signalling and gliotransmitter release in PD mice that result in altered synaptic function (Nanclares et al., 2023).

During the initial stages of disease pathology, astrocytes play a neuroprotective role by facilitating phagocytosis, maintaining proteostasis, and reducing extracellular inflammatory responses (Morales et al., 2017; Yang et al., 2022). Indeed, a recent report identified a subpopulation of astrocytes positive for Vitamin D activating enzyme which might be neuroprotective and beneficial in mitigating disease pathology (Mazzetti et al., 2022). However, scientists have made efforts to comprehend and identify the intricate mechanisms by which astrocytes transform into pathological signatures. Some of the astrocytic characteristics, including impaired chaperone-mediated autophagy, macroautophagy (di Domenico et al., 2019), disruption of Ca^2+^ signalling, reactive phenotypes, altered metabolic functions such as reduced glycolysis (Sonninen et al., 2020), pro-inflammatory responses and regional heterogeneity (Kostuk et al., 2019; Basurco et al., 2023) are considered as pathological switching traits of astrocytes in disease causation and progression and therefore targeting these pathways hold great therapeutic opportunity.

### Huntington’s disease (HD)

HD is an inherited neurodegenerative disorder with hallmarks such as progressive motor, cognitive and psychiatric dysfunction (Paulsen et al., 2001; Tabrizi et al., 2013) caused by an increased polyglutamine (PolyQ)-encoding CAG repeat (>36) in exon 1 of the huntingtin gene (HTT) (MacDonald et al., 1993). Both cortico-striatal and thalamo-cortical neural circuits are significantly affected in HD (Eidelberg and Surmeier, 2011). Rodent studies suggest that the accumulation of the mutant huntingtin protein (mHTT) is a direct contributor to HD disease pathology (Bradford et al., 2009). A recent study indicated that expression of mHTT specifically in astrocytes can worsen the disease progression, but still requires mHTT expression in neuronal cells to induce neurodegeneration (Jing et al., 2021). Additionally, engrafting mHTT expressing human glial progenitor cells into healthy mice elicited characteristics of HD (Benraiss et al., 2016). Conversely, reducing the accumulation of mHTT in astrocytes improved motor functions, decreased neuropsychiatric features and restored NMDA receptor function of striatal medium spiny neurons (MSNs) in the BACHD mice model of HD, therefore, highlighting the critical role of astrocytes in HD pathology (Wood et al., 2019).

Several astrocytic dysfunctions are associated with HD pathogenesis, such as low expression of glutamate transporters (Faideau et al., 2010), increased synthesis and release of astrocytic glutamate (Lee et al., 2013), and reduced extracellular glutamate uptake rate in the cortex and striatum (Lievens et al., 2001; Shin et al., 2005). Recently, reduced expression of astrocytic K_ir_ 4.1 channels was reported in striatal astrocytes, causing elevated levels of extracellular K^+^ ions and thereby increasing the excitability of MSNs (Tong et al., 2014). Moreover, glutamate transporter GLT-1 activity and its impact on synaptic currents appears to be dependent on Kir4.1 conductivity, which is perturbed in the HD mice model (Dvorzhak et al., 2016). However, these phenotypes, including the aberrant K^+^ ion levels, MSNs excitability profile and motor deficits were restored via viral delivery of K_ir_4.1 channels, emphasising the critical role of K^+^ homeostasis in HD (Tong et al., 2014). In line with this study, Diaz- Castro and colleagues deciphered the link between astrocytic Ca^2+^ signalling, GLT-1, and K_ir_4.1 in HD mice model and found that loss in homeostatic functions of GLT-1 and K_ir_4.1 led to aberrant glutamate and Ca^2+^ signalling altering striatal MSNs (Jiang et al., 2016). In addition, mHTT astrocytes exhibit reduced cholesterol synthesis which does not support proper synaptic and neuronal functioning (Valenza et al., 2010; Valenza et al., 2015). Moreover, altered brain energy metabolism such as reduced glucose uptake is also evident in HD, indicating that astrocytic-neuronal cross-talk can aid in the early detection of the disease (Boussicault et al., 2014). Supporting proteomics studies revealed compromised astrocytic metabolism, and impaired glutamate/GABA-glutamine cycle causing disruptions in the synthesis and release of glutamine and GABA in HD. Subsequently, Garcia and colleagues investigated the electrophysiological properties of astrocytes derived from Huntington patients’ iPSCs. They reported longer astrocytic spontaneous Ca^2+^ signals, impaired K^+^ inward rectifying currents, lower cell membrane capacitance and the inability of astrocytes to shield neurons from glutamate excitotoxicity, indicating that HD astrocytes are not capable to provide enough support for the neuronal population to thrive (Garcia et al., 2019). Interestingly, a recent study employed a transcriptomics approach to decipher the factors that drive astrocytic dysfunction in HD discovered an inverse relationship between the length of the PolyQ tail and metabolic activity, whereas astrocytic reactivity and DNA damage remained a constant factor (Lange et al., 2023).

### Amyotrophic lateral sclerosis (ALS)

ALS is a type of motor neuron disease (MNs) that is characterized by the gradual loss of upper and lower motor neurons which are responsible for muscle movement, speech, and breathing (Hardiman et al., 2017; Buskila et al., 2019b). Astrocytes have been held accountable for causing MN death in ALS via numerous mechanisms, including the release of soluble neurotoxic factors that contribute to the selective death of MNs (Nagai et al., 2007). Among these, lipocalin 2, an inducible factor that is produced by astrocytes having a mutant TAR DNA-binding protein 43 (TDP-43), RNA-binding proteins fused in sarcoma (FUS) genes, and inorganic polyphosphate (polyp) secreted by mutant SOD1, TARDBP, and C9ORF72 astrocytes, are known to selectively eradicate MNs (Bi et al., 2013; Kia et al., 2018; Arredondo et al., 2022). Moreover, neutralisation of TNF-α in mtFUS mice model for ALS or expression of mFUS in astrocytes of TNF-α KO mice did not exhibit motor dysfunctions and prevented MN death suggesting TNF-α as a potential therapeutic target (Jensen et al., 2022). This demonstrates that astrocytes secrete different neurotoxic factors according to the mutations they carry. Consistent with these studies, treatment of primary spinal culture with astrocytic culture medium from SOD1 mice (ACM-hSOD1^G93A^) led to elevated persistent sodium inward currents, increased intracellular Ca^2+^ transients, and hyperexcitability of MNs that ultimately result in their death (Fritz et al., 2013). In addition, dysfunctional astrocytic glutamate transporters, elevated [K^+^]_o_ and glutamate concentrations make the MNs vulnerable to excitotoxicity and worsen the disease progression (Rothstein et al., 1995; Rothstein et al., 1996; Do-Ha et al., 2018).

Recently, metanalysis of astrocytes from ALS mice and patient-derived iPSCs suggested that ALS- astrocytes are characterised by overexpression of genes implicated in immune system response and endoplasmic reticulum stress, and reduced expression of genes that affect glutamate uptake, maintenance of synaptic integrity, and support to neurons. These characteristics can be considered as priming factors which aid astrocytes to achieve reactive and pro-inflammatory detrimental phenotypes (Ziff et al., 2022). In line with this study, specific knock-out of activation factors in astrocytes, such as IL-1α, TNFα, and C1q prolonged the survivability of SOD1^G93A^ mice (Guttenplan et al., 2020). Interestingly, the expression of astrocytic neurotoxic factor TNF-α is dependent on the activation of NF-κB, which plays a critical role in determining disease progression, as its activation in the presymptomatic stage makes the astrocytes acquire neuroprotective traits, while in the symptomatic stage, its activation worsens the disease (Ouali Alami et al., 2018). Indeed, mutant astrocytes are considered to cause higher levels of oxidative stress and dysregulated autophagy (Madill et al., 2017; Birger et al., 2019). Furthermore, malfunctioning of astrocytic metabolism, such as reduced NADH and adenosine deaminase production (Allen et al., 2019), transformed adenosine, fructose and glycogen metabolism and impaired lactate shuttling (Madji Hounoum et al., 2017) are evident in mutant ALS astrocytes, which result in energy deprivation and starvation in both astrocytes and neurons. This can be related to the accelerated senescence of astrocytes in ALS due to the shutdown of astrocytic support required to maintain healthy neuronal function and prevent MN death (Das and Svendsen, 2015).

## Conclusion

Traditionally, most research about neural signaling took a neuro-centric approach, focusing on neuronal dysfunction, connectivity, and morphology. Our understanding of the role astrocytes play in the modulation of neuronal signalling has come a long way in the past two decades, mainly due to the development of new techniques, including two-photon laser scanning microscopy, electron microscopy reconstruction, genetically encoded Ca^2+^ dyes (GCaMPs), viral vector delivery systems, optogenetic and chemogenetic tools (Goshi et al., 2020; Delgado and Navarrete, 2023). Neuronal-astrocytic interactions are complex, mainly due to the expression of many receptors and channels in both neurons and astrocytes. The fact that astrocytic heterogeneity changes between different brain regions and during ageing pose another layer of complexity. Thus, disecting specific astrocytic processes that affect neuronal activity raise limitations. To overcome these limitations, the field should focus on developing new techniques and tools that can be used *in situ* to target specific astrocytic channels and proteins in specific brain areas, as recently reviewed by (Yu et al., 2020). Indeed, emerging tools such as the recently developed ‘neuron-astrocyte proximity assay (NAPA) by Khakh group (Octeau et al., 2018) and the genetically encoded K^+^ indicators by Dong group (Shen et al., 2019) have great potential is securing progress in this field (Gao et al., 2016; Yu et al., 2020).

Neuronal astrocytic interactions are highly dynamic and span a wide spectrum ranging from molecular to network levels. While some of the impacts astrocytes convey on neuronal signalling are carried via direct pathways, including downregulation of synaptic receptors and LTP, other astrocytic processes, such as heterosynaptic depression and regulation of hyper-synchronised activity of neurons are carried indirectly. Elucidating the involvement of astrocytic dysfunction during ageing and neurodegeneration has great potential to develop future CNS-related therapeutic targets. Indeed, as astrocytes are dividing cells and more adaptable than neurons, therapies aimed at astrocytic dysfunction rather than at neurons may prove superior in treating CNS disorders.
